# The impact of expiratory muscle strength training on voluntary cough effectiveness in Huntington's disease

**DOI:** 10.1111/ene.16500

**Published:** 2024-09-30

**Authors:** Romana Konvalinkova, Martin Srp, Kristyna Doleckova, Vaclav Capek, Ota Gal, Martina Hoskovcova, Radim Kliment, Jan Muzik, Evzen Ruzicka, Jiri Klempir

**Affiliations:** ^1^ Department of Neurology and Centre of Clinical Neuroscience General University Hospital and First Faculty of Medicine, Charles University Prague Czech Republic; ^2^ Faculty of Biomedical Engineering Czech Technical University Prague Czech Republic

**Keywords:** cough, Huntington disease, mHealth, resistance training, respiratory muscles

## Abstract

**Background and purpose:**

Dysfunction of the airway defence system in Huntington's disease (HD) is a significant but often overlooked problem. Although expiratory muscle strength training (EMST) is frequently utilized in cough effectiveness treatment, its specific impact in HD patients has not yet been explored. This study investigated the effects of EMST on voluntary peak cough flow (vPCF) in HD patients and evaluated the retention of potential gains post‐intervention.

**Methods:**

In this prospective case‐controlled trial, 29 HD patients completed an 8‐week wait‐to‐start period, which served to identify the natural development of expiratory muscle strength and vPCF. This was followed by 8 weeks of EMST training and an additional 8 weeks of follow‐up. The study's outcome parameters, vPCF and maximum expiratory pressure (MEP), were measured against those of age‐ and sex‐matched healthy controls.

**Results:**

Huntington's disease patients had significantly lower MEP (*p* < 0.001) and vPCF (*p* = 0.012) compared to healthy controls at baseline. Following the EMST, significant improvements in MEP (*d* = 1.39, *p* < 0.001) and vPCF (*d* = 0.77, *p* = 0.001) were observed, with HD patients reaching the cough performance levels of healthy subjects. However, these gains diminished during the follow‐up, with a significant decline in vPCF (*d* = −0.451, *p* = 0.03) and in MEP (*d* = −0.71; *p* = 0.002).

**Conclusions:**

Expiratory muscle strength training improves expiratory muscle strength and voluntary cough effectiveness in HD patients, but an ongoing maintenance programme is necessary to sustain the improvements.

## INTRODUCTION

Dysfunction of the airway defence system in Huntington's disease (HD) is a significant but often overlooked problem, which can lead to aspiration pneumonia, a major reason for hospital admissions [1] and the primary cause of death amongst these patients [2]. The airway defence system comprises a continuum of highly coordinated behaviours, ranging from swallowing (a primary preventative behaviour) to coughing (a primary corrective behaviour) [3]. Previous studies have identified impairments in both swallowing and coughing amongst HD patients, including those at mid‐stages of the disease who already face clinically significant challenges with regard to these key components of the airway defence system [4, 5].

Expiratory muscle strength training (EMST) has been found to improve the airway defence system across various clinical populations, including patients with Parkinson's disease (PD) [6], multiple sclerosis [7], amyotrophic lateral sclerosis [8] and stroke [9]. EMST is an exercise‐based treatment utilizing a calibrated device equipped with a one‐way, spring‐loaded valve that is designed to enhance the expiratory muscles' strength through overload. Since the strength of these muscles is important for generating adequate expiratory flow pressure, EMST is frequently utilized for improving cough effectiveness, as measured by voluntary peak cough flow (vPCF) [10]. If the significantly reduced respiratory muscle strength in HD patients compared to healthy individuals [4, 11, 12] is considered, it can be hypothesized that the EMST should also be effective in enhancing vPCF in HD patients.

Nevertheless, research on the effects of respiratory muscle strength training in HD patients is limited, with only two studies addressing this topic [4, 13]. In a pilot randomized controlled trial, Reyes et al. [13] demonstrated that 4 months of progressive inspiratory and expiratory strength training was feasible and safe for HD patients. The patients improved their maximal inspiratory and expiratory pressures, as well as their forced vital capacity and peak expiratory flow. The second study, a pilot pseudo‐randomized controlled trial, found a modest effect on vPCF after inspiratory muscle strength training when data from both the experimental and the control group were pooled [4]. Thus, the specific impact of EMST on vPCF in HD patients remains unexplored.

Another important aspect that has been emphasized in light of the follow‐up results of prior PD research is the strong need for a sustained EMST programme [14]. The study by Troche et al. [14] highlighted the need for EMST maintenance programmes to sustain the benefits to the airway defence system following intensive periods of training. Thus, understanding the EMST follow‐up period in HD patients is also crucial.

The aim of this study was to investigate the effects of the EMST protocol on vPCF in HD patients and the retention of potential gains after the end of the intervention. It is hypothesized that (1) the EMST protocol would lead to increased vPCF in HD patients and (2) these gains would be retained, albeit diminished, during the follow‐up period.

## METHODOLOGY

### Subjects

The study was approved by the Ethics Committee of the General University Hospital in Prague (93/21S‐IV). Informed consent was obtained from all participants before enrolment in this study. Participants were recruited from the Movement Disorders Centre, Department of Neurology, General University Hospital in Prague, between January 2022 and May 2023. The inclusion criteria were (1) diagnosis of HD, confirmed by genetic testing; (2) age ≥18 years; (3) stable medication regimen for 4 weeks before the initiation and throughout the trial. The exclusion criteria were (1) other concomitant neurological disorders that were relevant to the aim of the study; (2) a history of cardiovascular or lung disease; and (3) severe cognitive impairment (Mini‐Mental State Examination <19). Given the importance of respiratory muscles for the production of sufficient expiratory pressure and thus an effective expiratory flow to clear airway secretions, Reyes et al. [13] recommend calculating the sample size in relation to the maximum respiratory pressures in future HD studies. Based on data from their study [13], a power analysis showed that a sample size of 24 participants would provide 80% power to detect, with an *α* of 0.05, a post‐treatment difference of 25 (±35) cmH_2_O in maximum expiratory pressure (MEP). To accommodate an attrition rate of 20%, it was calculated that the sample size should be 29 participants. The subjects with HD who were included were compared to age‐ and sex‐matched healthy subjects recruited from the hospital staff and their relatives.

### Study design

**FIGURE 1:**
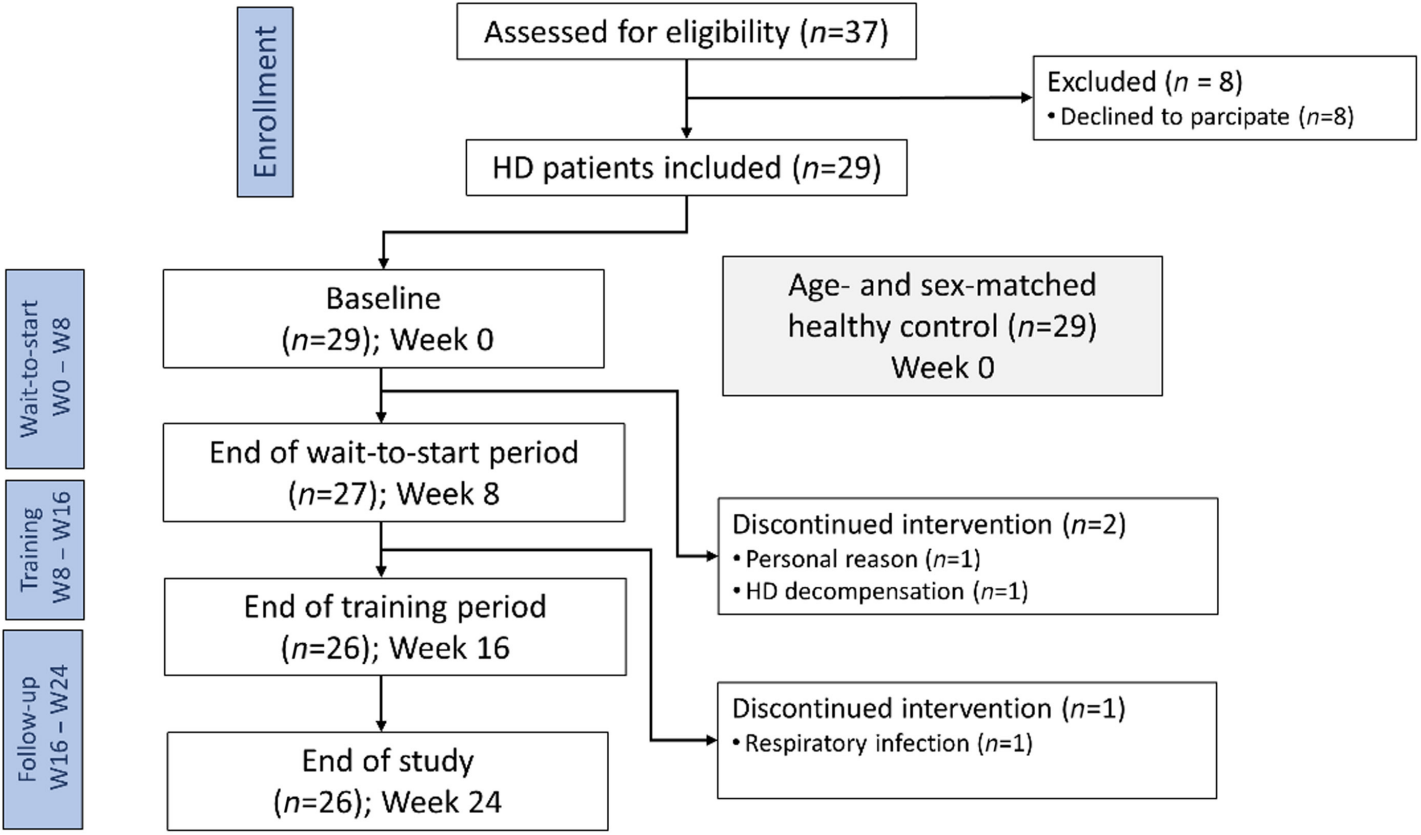
Flowchart of the HD patients in the study. EMST, Expiratory muscle strength training; HD, Huntington's disease; W, Week.

Participants in this prospective case‐controlled study completed an 8‐week wait‐to‐start period, which served to identify the natural development of expiratory muscle strength and vPCF. This was followed by 8 weeks of EMST training and an additional 8 weeks of follow‐up. In total, participants completed four visits (Figure 1).

### Study outcome parameters

The vPCF was selected as the primary outcome of cough efficacy, due to its established correlation with the capacity to expel aspirated substances from the airway [15]. MEP was selected as the primary outcome measure of respiratory strength [10]. The primary study aim was to produce a change in the vPCF after the 8‐week EMST (W8 vs. W16). The secondary study aim was to produce a change in the MEP changes (W8 vs. W16 and W16 vs. W24) in the vPCF after an 8‐week follow‐up (W16 vs. W24). Healthy controls completed the same measurements, but only at baseline. Values obtained from healthy controls were taken as normative data.

### Assessment visits

The same assessment protocol, which included vPCF and MEP assessments, was completed at pre and post wait‐to‐start periods, post‐treatment and at 8‐week follow‐up. Each visit took place at the Department of Neurology and the Centre of Clinical Neuroscience at the General University Hospital and the First Faculty of Medicine, Charles University in Prague, at the same time of day. Assessment visits were completed by a trained research physiotherapist with experience conducting vPCF and MEP measurements on a daily basis.

The vPCF was measured using a pneumotachograph (BTL Cardiopoint Spiro, BTL Industries), which meets the recommendations of the American Thoracic Society and the European Respiratory Society for range and accuracy in forced expiratory manoeuvres [16]. The assessments were conducted according to established guidelines [17], with the subjects seated and wearing nose clips to prevent air leakage through the nose. Participants were instructed to cough forcefully into a mouthpiece connected to a pneumotachograph after a full inhalation, with verbal encouragement provided throughout the process. To ensure accuracy, at least three measurements with less than 5% variation between them were taken, and the highest value was recorded as the vPCF.

Maximum expiratory pressure assessments were performed using a flanged rubber mouthpiece connected to a pressure manometer (Micro RPM, Vyaire Medical). MEP assessments were conducted according to the statements on respiratory muscle testing of the American Thoracic Society and the European Respiratory Society [17]. Participants were seated upright and instructed to inhale deeply before exhaling forcefully into the manometer. Nose clips were applied to prevent air leakage through the nose. Participants received verbal encouragement throughout the process. To ensure consistency, at least three measurements with less than 10% variation between them were taken, and the highest value was recorded as the MEP.

### Expiratory muscle strength training

Participants followed the expiratory muscle training programme using an expiratory muscle trainer (EMST75 or EMST150; Aspire Products, LLC, USA) with a one‐way, spring‐loaded pressure relief valve, which provided a pressure threshold range from 5 to 150 cmH_2_0. The adjustable spring allows for discrete changes in the valve blocking the flow of air until sufficient expiratory pressure is produced, thus modifying the physiological load placed on the muscles. EMST therapy sessions were completed at home on five self‐selected days per week. Participants were instructed to perform five sets of five forceful expirations per session over an 8‐week period. This design was based on the study by Reyes et al. [18]. The training device was set at 75% of each participant's MEP value. After 4 weeks of EMST, the MEP values were reassessed in order to readjust the training difficulty to 75% of each participant's current MEP. This design was based on the Pitts et al. study [19]. Following the recommendation of a meta‐analysis [20], which emphasized enhanced supervision and support for exercise programmes as critical factors in facilitating adherence and optimizing outcomes in HD patients, the EMST is combined with a smartphone‐based visual feedback application (SpiroGym app) [21] to increase patient motivation, adherence and training effort. Additionally, a research physiotherapist provided bi‐weekly coaching to participants over the phone.

### SpiroGym application

All participants were equipped with smartphones containing the SpiroGym app, which evaluates real‐time training data using a microphone attached to an expiratory muscle trainer. When the expiratory manoeuvre is correctly performed using the expiratory muscle trainer, the exhalation valve opens and the air flow increases the sound level detected by the microphone. Simultaneously, the app gives visual feedback on the smartphone screen in real time using a curve that depicts the current sound level. The patient's task is to keep the curve in the training zone. Further technical details about the SpiroGym app are provided in the study by Srp et al. [21]. The app also allows the patient to check training data from previous workouts and monitor long‐term developments. The SpiroGym app proved to be feasible and useful in patients with PD, including those with mild cognitive impairment [21].

### Statistical analysis

Differences in baseline characteristics between HD patients and healthy controls were analysed using a paired *t* test in the case of age, weight and height; a paired Wilcoxon test in the case of body mass index, MEP and vPCF; and a chi‐squared test in the case of sex. The effects of the EMST were calculated in terms of differences between the trend for the training/follow‐up period and the trend for the wait‐to‐start period (difference of differences). Cohen's *d* was used to evaluate the corresponding effect sizes and the paired Wilcoxon test to evaluate its significance. *p* values were adjusted for multiple comparisons by the Holm method. *p* values of less than 5% were considered statistically significant. The analysis was performed using an R statistical package version 4.3.2.

## RESULTS

In total, 29 patients were enrolled in the study and compared with 29 age‐ and sex‐matched healthy controls. The difference in demographic and clinical characteristics between HD patients and the healthy controls was not statistically significant with the exception of a significantly lower MEP (*p* < 0.001) and vPCF (*p* = 0.012) amongst the HD patients. A summary of demographic and clinical characteristics is provided in Table 1. During the wait‐to‐start period (W0–W8), two patients discontinued the programme whilst another one dropped out during the training period (W8–W16). The patients' reasons for doing so are provided in Figure 1. The mean Unified Huntington's Disease Rating Scale Total Motor Score (UHDRS‐TMS) increased by 2.97 points over the 24‐week period. All HD patients were able to independently operate the SpiroGym app and reported no technical problems during the training period. No adverse effects of EMST were reported during the training period.

**TABLE 1:** Baseline demographic and clinical characteristics of the HD patients and healthy controls. [Correction added on 06 November 2024 after first online publication: Table 1 has been corrected in this version.]

	HD patients (*n* = 29)	Healthy controls (*n* = 29)	Group difference	*p*‐Value
Gender (female/male)	15/14	15/14		1.000
Age (years)	47.3 ± 12.0 (21–69)	47.7 ± 11.6 (26–67)	0.3 ± 2.6 (‐5–8)	0.522
Weight (kg)	75.3 ± 17.1 (44–115)	77 ± 14.8 (52 –105)	1.7 ± 15.0 (‐31–22)	0.545
Height (cm)	171.6 ± 11.1 (152–197)	172.1 ± 9.3 (155–190)	0.5 ± 9.3 (‐32–18)	0.766
BMI	25.6 ± 5.3 (17.4–36.7)	26.1 ± 4.3 (20.6–36.8)	0.5 ± 5.1 (‐8.8–16.4)	0.565
MEP (cmH_2_0)	91.6 ± 46.3 (28–196)	152.1 ± 46.6 (70–233)	60.5 ± 59.3 (‐86–196)	<0.001
vPCF (l/min)	433.4 ± 127.8 (213 –817)	501.6 ± 119 (294–745)	68.1 ± 125.0 (‐193–456)	0.012
Disease duration (years)	5.2 ± 3.4 (1–15)			
CAG repeat	44.2 ± 3.9 (40–54)			
UHDRS‐TMS	18.3 ± 11.5 (0–38)			
TFC	10.3 ± 2.5 (6–13)			
SF staging (*n*)				
Stage I	14 (48%)			
Stage II	13 (45%)			
Stage III	2 (7%)			
Stage IV, V	0			
IS	89.1 ± 9.9 (70–100)			
FA	22.1 ± 3.2 (15–29)			
MMSE	26.7 ± 2.7 (19–30)			

*Note*: Values are mean ± standard deviation (min.‐max.)

Abbreviations: BMI, body mass index; CAG, cytosine‐adenine‐guanine; FA, functional assessment; HD, Huntington's disease; IS, independence scale; MEP, maximum expiratory pressure; MMSE, Mini‐Mental State Examination; SF, Shoulson‐Fahr; TFC, total functional capacity; UHDRS‐TMS, Unified Huntington's Disease Rating Scale Total Motor Score; vPCF, voluntary peak cough flow.

### Voluntary peak cough flow

**FIGURE 2:**
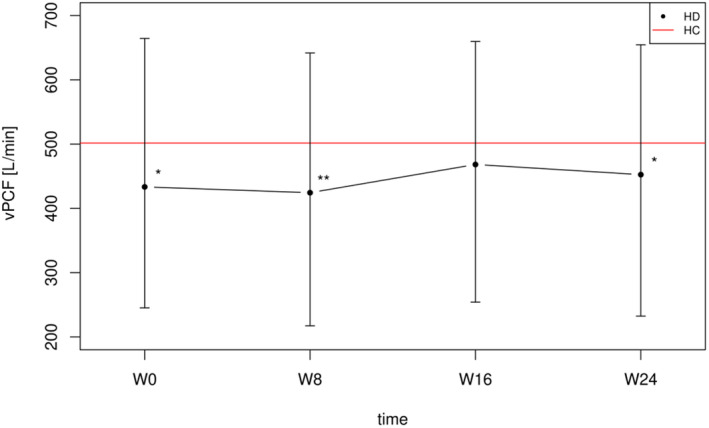
Development of HD patient vPCF values across the study period in comparison with healthy controls. *P* value (adjusted for multiple comparisons) of the difference between HD patients vPCF at particular timepoints and HC vPCF normative values. * *p*<0.05, ** *p*<0.01. HC, Healthy Controls; HD, Huntington Disease patients; vPCF, Voluntary Peak Cough Flow; W, Week.

No significant changes in vPCF (*d* = −0.32; *p* = 0.343) were observed after the wait‐to‐start period (W0–W8). The mean participant's adherence to the recommended expiratory manoeuvres during the training period (W8–W16) was 81% (810 manoeuvres out of 1000). During the training period (W8–W16), EMST had a medium effect on vPCF (*d* = 0.77; *p* = 0.001), resulting in a 10% increase. A significant decline in vPCF (*d* = −0.451; *p* = 0.03) was observed after the follow‐up period (W16–W24), with a decrease of 3.4%. A summary of the vPCF results is provided in Table 2. Throughout the study, a significant difference in vPCF was maintained between the HD patients and the healthy control group with the exception of the end of the training period (W16), where the difference was not statistically significant (*p* = 0.067), as shown in Figure 2.

**TABLE 2:** Changes in MEP and vPCF from baseline (W0) to end of the study (W24) in HD patients.

Time point	MEP (cmH_2_O)	vPCF (L/min)
Week 0 (baseline)	91.6 ± 46.3 (30.8–192.5)	433.4 ± 127.8 (245.2–664.4)
Week 8	85.3 ± 45.1 (36.4–189.9)	424.4 ± 117.9 (217.3–641.8)
∆W8–W0	−5.19 ± 16.2 (−40.7–18.4)	−15.2 ± 51.4 (−150.5–43.3)
*p* value of ∆	0.248	0.343
Effect size (*d*)	−0.32 (−1.12–0.48)	−0.30 (−1.09–0.50)
Week 16	122.0 ± 55.1 (57.5–232.1)	468.3 ± 111.9 (254.1–659.7)
∆W16–W8	35.1 ± 24.7 (−7.4–78.1)	43.7 ± 45.3 (−36.6–107.7)
Pure effect of EMST*	40.2 ± 28.9 (3.0–101.6)	58.3 ± 75.3 (−73.6–201.1)
*p* value of pure effect	<0.001	0.001
Effect size (*d*)	1.39 (0.49–2.29)	0.77 (−0.06–1.61)
Week 24	116.4 ± 52.8 (54.4–227.5)	452.5 ± 119.7 (232.4–654.5)
∆W24–W16	−5.6 ± 7.8 (−21.4–5.8)	−15.8 ± 35.0 (−73.0–49.1)
*p* value of ∆	0.002	0.030
Effect size (*d*)	−0.71 (−1.55–0.12)	−0.45 (−1.27–0.37)

*Note*: Values are mean ± standard deviation (95% confidence interval).

Abbreviations: EMST, expiratory muscle strength training; HD, Huntington’s disease; MEP, maximum expiratory pressure; vPCF, voluntary peak cough flow; W, week; Δ, change.

* The pure effect of EMST on the observed variables (training Δ) was computed as the change of the variable during the training period, that is, (W16 value ‐ W8 value), with the change of the variable during the wait‐to‐start period, that is, (W8 value ‐ W0 value), subtracted. [Correction added on 05 November 2024 after first online publication: Table 2 Note has been revised in this version.]

### Maximum expiratory pressure

**FIGURE 3:**
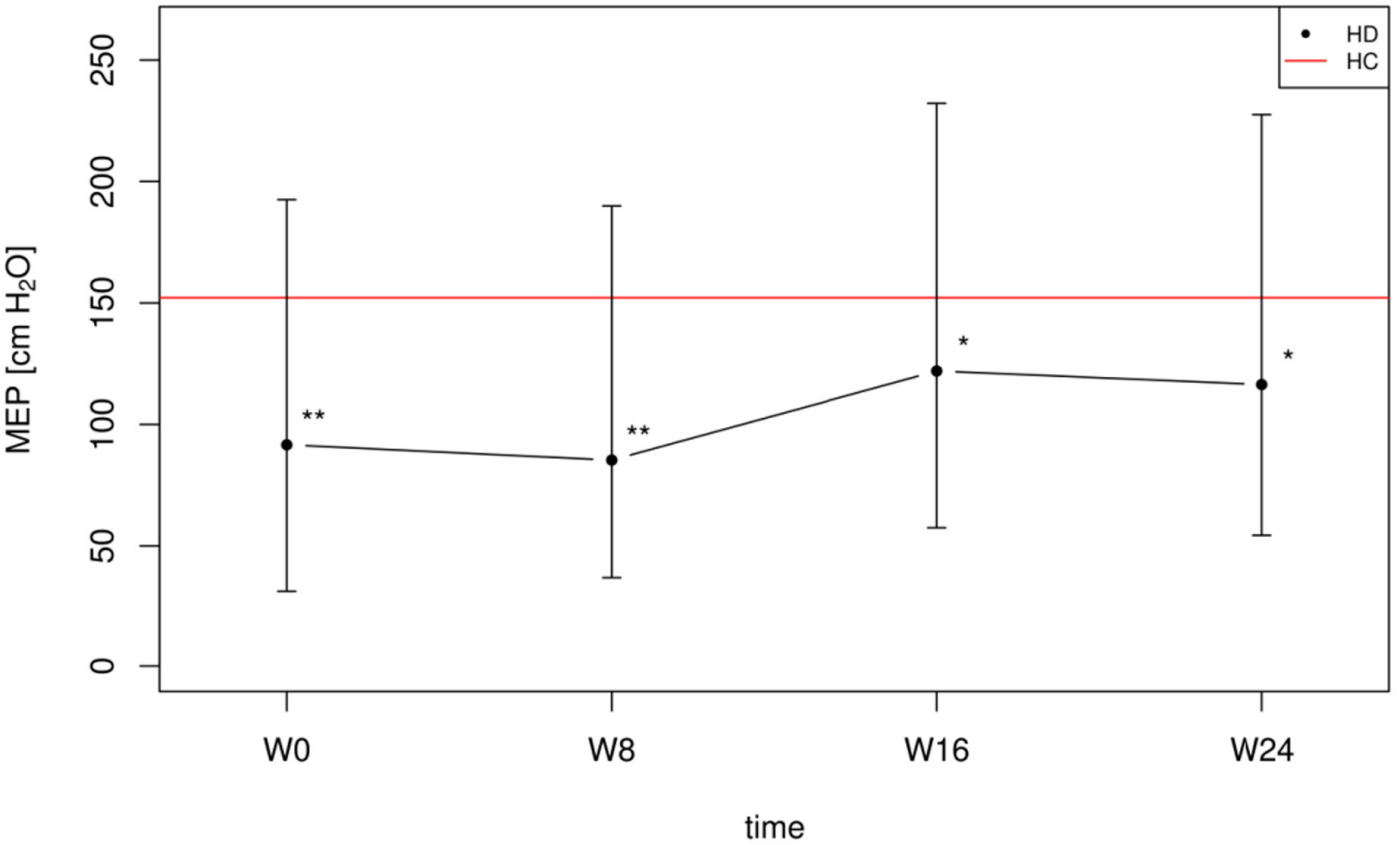
Development of HD patient MEP values across the study period in comparison to healthy controls. *P* value (adjusted for multiple comparisons) of the difference between HD patients' MEP at the study timepoints and HC' MEP normative values. * *p*<0.05, ** *p*<0.001. HC,Healthy Controls; HD,Huntington Disease patients; MEP,Maximum Expiratory Pressure; W, Week.

Following the wait‐to‐start period (W0–W8), no significant change was observed in MEP (*d* = −0.32; *p* = 0.248). During the training period (W8–W16), EMST had a large effect on the participants’ MEP (*d* = 1.39; *p* < 0.001), whilst a significant decline in MEP (*d* = −0.71; *p* = 0.002) was observed after the follow‐up period (W16–W24). A summary of MEP results is provided in Table 2. The difference in MEP between the HD and healthy controls group remained significant throughout the entire study period (Figure 3).

## DISCUSSION

This is the first study to test the effects of an EMST protocol on cough efficacy in HD patients and explore the retention of changes after the end of training. The most important and clinically relevant finding of this study demonstrates that, despite ongoing neurodegeneration, HD patients exhibited significant increases in vPCF and MEP following an 8‐week EMST protocol. Moreover, after EMST, patients attained the vPCF levels of healthy controls, but these gains significantly diminished post follow‐up. The design of our study, which incorporated a wait‐to‐start phase, had a unique advantage in so far as it enabled us to observe the natural decline of vPCF and MEP in HD patients and to clearly identify the pure effects of EMST.

The observed 2.97‐point increase in UHDRS‐TMS was consistent with the HD progression seen in previous studies [22, 23]. EMST was not expected to impact UHDRS‐TMS. Whilst it is acknowledged that the progression of HD is neither linear nor constant throughout the lifespan, it is believed that the natural progression of vPCF and MEP during this study was valid due to consistent declines observed in both non‐training periods (W0–W8 and W16–W24). The vPCF results showed that 8 weeks of EMST had a moderate effect (*d* = 0.77) on vPCF enhancement. On average, the HD patients improved their vPCF from 424.4 to 468.3 L/min (a 10% improvement). These gains are clinically meaningful, considering that before EMST there was a significant difference in vPCF levels between patients and their age‐ and sex‐matched healthy controls, which was no longer evident post‐training. In the only other published HD patient study, Jones et al. documented a smaller effect (*d* = 0.37) on vPCF following a 6‐week programme of inspiratory muscle training compared to our study [4]. This difference is in line with previous research showing that EMST is more beneficial than an inspiratory muscle training programme for improving vPCF [18]. Additionally, the findings of our study align with previous EMST research in the PD population, where improvements in vPCF ranged from 6% to 15.6% [10, 18, 24]. Another key contribution of this study was to examine the retention of vPCF following an 8‐week follow‐up period. vPCF declined significantly after this period (by 3.4%), causing the vPCF levels to once again significantly differ from those of the healthy controls. This finding highlights the need for the development of maintenance programmes to sustain the benefits of intensive training periods. According to Sapienza and Hoffman [25], performing 25 expiratory manoeuvres twice a week is considered the minimum frequency necessary for an effective maintenance period. However, whether this frequency is sufficient to maintain benefits in HD patients needs to be confirmed in future studies.

Another important question concerns the optimal timing for initiating respiratory training in the progression of the disease. According to a survey cited in the clinical recommendations for physiotherapy in HD [26], 48.9% of clinicians report using respiratory exercises to treat individuals in the late stages of HD. However, incorporating respiratory exercises into the treatment plan for mid‐stage HD patients, who already face significant challenges in cough effectiveness compared to healthy individuals, might offer more substantial benefits. Patients in the mid‐stage of HD are generally less cognitively impaired, facilitating more effective participation in EMST training. This advantage, combined with the efficacy of such training as demonstrated in this study, suggests that earlier intervention could enhance therapeutic outcomes and potentially decelerate the deterioration in cough efficacy.

EMST has as its primary treatment target to produce an increase in MEP in order to improve expiratory force generation. The only previously published study reported a 14.5% increase in MEP amongst HD patients after 2 months of EMST [13]. However, our study demonstrated a larger effect on MEP, with HD patients improving their MEP from 85.3 to 122 cmH_2_O (a 43% improvement). This discrepancy might be attributed in part to the small sample size (*n* = 9) of the previous study, which could have limited the robustness of their results. Additionally, the lower baseline MEP values in our cohort suggest that our patients had a greater scope for improvement. Moreover, the added value of the visual feedback provided by the SpiroGym app may account for the better results obtained in our study, since this feedback potentially enhances the EMST and amplifies its effects on clinical outcomes. Such effect has been mentioned in previous studies [21, 27].

The home‐based respiratory muscle training was well tolerated by the participants, with 81% mean adherence rates and no reports of adverse events. The adherence rate from our study corresponds closely with findings from a systematic review and meta‐analysis on the effects of home‐based exercise in HD, which reported an average adherence of 78% [28].

The interpretation of this study's results should take into account its limitations. The first was the lack of a control group. A future fully powered randomized controlled trial is required to validate our findings. Second, this study predominantly focused on early to mid‐stage HD patients who had relatively intact cognitive functions, leaving a gap in our understanding of whether EMST is applicable to late‐stage HD patients. Third, it did not select the HD patients based on their baseline primary outcome results. This approach may have influenced the observed training effects, especially in patients who initially had higher MEP and vPCF levels, leading to a ceiling effect. Fourth, the healthy control group for vPCF and MEP was assessed only once, limiting insight into their variation. Therefore, future comparative research should validate our results and determine whether EMST is applicable to late‐stage HD patients, especially those with cognitive impairment. Additionally, it should investigate additional important related variables including quality of life, hospitalization rates and aspiration pneumonia rates.

Overall, this study is the first to demonstrate the effectiveness of EMST as a non‐pharmacological intervention to enhance cough effectiveness in HD patients. However, the decline in benefits following the end of the intervention highlights the need for a maintenance programme to preserve the therapeutic gains.

## AUTHOR CONTRIBUTIONS

**Romana Konvalinkova:** Conceptualization; investigation; methodology; writing – original draft; writing – review and editing; project administration; visualization. **Martin Srp:** Conceptualization; methodology; writing – review and editing; project administration; investigation; validation; visualization. **Kristyna Doleckova:** Project administration; writing – review and editing; conceptualization. **Vaclav Capek:** Formal analysis; writing – review and editing; data curation. **Ota Gal:** Conceptualization; writing – review and editing; methodology. **Martina Hoskovcova:** Conceptualization; methodology; writing – review and editing. **Radim Kliment:** Software; writing – review and editing. **Jan Muzik:** Software; writing – review and editing. **Evzen Ruzicka:** Conceptualization; methodology; writing – review and editing; supervision; resources. **Jiri Klempir:** Conceptualization; methodology; writing – review and editing; funding acquisition; supervision; resources.

## FUNDING INFORMATION

The research underlying this article was supported by the MH CZ–DRO–VFN00064165.

## CONFLICT OF INTEREST STATEMENT

The authors declare that there are no conflicts of interest with regard to the research relating to the manuscript.

## Data Availability

The underlying data used in this study are available from the corresponding author upon reasonable request.
